# Three-Dimensional Printing Constructs Based on the Chitosan for Tissue Regeneration: State of the Art, Developing Directions and Prospect Trends

**DOI:** 10.3390/ma13112663

**Published:** 2020-06-11

**Authors:** Farnoosh Pahlevanzadeh, Rahmatollah Emadi, Ali Valiani, Mahshid Kharaziha, S. Ali Poursamar, Hamid Reza Bakhsheshi-Rad, Ahmad Fauzi Ismail, Seeram RamaKrishna, Filippo Berto

**Affiliations:** 1 Department of Materials Engineering, Isfahan University of Technology, Isfahan 84156-83111, Iran; farnoosh.pahlevanzadeh@gmail.com (F.P.); remadi@cc.iut.ac.ir (R.E.); ma.kharaziha@gmail.com (M.K.); 2 Department of Anatomical Science, School of Medicine, Isfahan University of Medical Sciences, Isfahan, Iran; valiani@med.mui.ac.ir; 3 Biomaterials, Nanotechnology, and Tissue Engineering Group, Advanced Medical Technology Department, Isfahan University of Medical Sciences, Isfahan 81746-73461, Iran; ali.poursamar@amt.mui.ac.ir; 4 Advanced Materials Research Center, Department of Materials Engineering, Najafabad Branch, Islamic Azad University, Najafabad, Iran; 5 Advanced Membrane Technology Research Center (AMTEC), Universiti Teknologi Malaysia, Skudai 81310, Johor Bahru, Johor, Malaysia; afauzi@utm.my; 6 Department of Mechanical Engineering, National University of Singapore, 9 Engineering Drive 1, Singapore 117576, Singapore; seeram@nus.edu.sg; 7 Department of Mechanical and Industrial Engineering, Norwegian University of Science and Technology, 7491 Trondheim, Norway

**Keywords:** 3D printing, chitosan, bio-inks, scaffolds, fabrication process, drug delivery, tissue engineering, biomedical applications

## Abstract

Chitosan (CS) has gained particular attention in biomedical applications due to its biocompatibility, antibacterial feature, and biodegradability. Hence, many studies have focused on the manufacturing of CS films, scaffolds, particulate, and inks via different production methods. Nowadays, with the possibility of the precise adjustment of porosity size and shape, fiber size, suitable interconnectivity of pores, and creation of patient-specific constructs, 3D printing has overcome the limitations of many traditional manufacturing methods. Therefore, the fabrication of 3D printed CS scaffolds can lead to promising advances in tissue engineering and regenerative medicine. A review of additive manufacturing types, CS-based printed constructs, their usages as biomaterials, advantages, and drawbacks can open doors to optimize CS-based constructions for biomedical applications. The latest technological issues and upcoming capabilities of 3D printing with CS-based biopolymers for different applications are also discussed. This review article will act as a roadmap aiming to investigate chitosan as a new feedstock concerning various 3D printing approaches which may be employed in biomedical fields. In fact, the combination of 3D printing and CS-based biopolymers is extremely appealing particularly with regard to certain clinical purposes. Complications of 3D printing coupled with the challenges associated with materials should be recognized to help make this method feasible for wider clinical requirements. This strategy is currently gaining substantial attention in terms of several industrial biomedical products. In this review, the key 3D printing approaches along with revealing historical background are initially presented, and ultimately, the applications of different 3D printing techniques for fabricating chitosan constructs will be discussed. The recognition of essential complications and technical problems related to numerous 3D printing techniques and CS-based biopolymer choices according to clinical requirements is crucial. A comprehensive investigation will be required to encounter those challenges and to completely understand the possibilities of 3D printing in the foreseeable future.

## 1. Introduction

**Figure 1:**
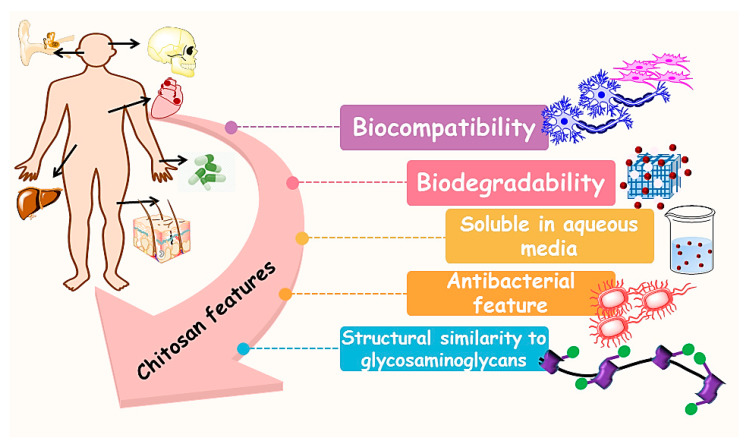
Suitable chitosan features for biomedical application.

Lesions and disorders that need tissue as well as organ transplantation continue to be important issues in clinical medicine, and there are also issues concerning the use of current methods which consist of auto-transplantation and xeno-transplantation. Thus, one can find substantial restrictions. Donor deficiency regarding organ transplantations tends to be a significant clinical problem globally [1,2]. Possible challenges which are undoubtedly experienced with conventional approaches consist of difficulties, extra injuries, restricted source donors, and immunological rejection. However, the implantation of synthetic organs in clinical treatments is frequently effective and enhances the sufferers’ quality of life. These synthetic organs are usually created from synthetic and natural polymers, ceramics, metals, and composites [1,2]. Concerning these kinds of materials, natural-based polymers tend to be considered among the large number of ubiquitous types of components in the medical related field [3]. Due to the fact that they occur in nature, components of both plant and animal origin are typically assumed to display an improved compatibility with human hosts, a capability to present bioactivity and biodegradation. Consequently, natural nano-materials and particulate components can demonstrate these features in circumstances in which synthetic components have never fulfilled medical objectives [1,2,3]. Natural polymers present in nature are usually gathered directly into six primary categories with regard to their resources: proteins, polysaccharides polynucleotides, polyisoprenes, polyesters, and lignin [4]. One of the polysaccharides which is made from shells of aquatic animals is chitin. CS, the main derivative of chitin, dates back to 1859 based on a study conducted by Rouget. The term CS was initially presented in 1894 by Hoppe-Seyler [5]. Chitosan as a biodegradable polymer has drawn considerable attention for biomedical purposes such as nano-fibers for wound dress applications, drug release systems, and space fillers [6,7]. Figure 1 exhibits the 3D printing concept and characteristic for augmentation of chitosan 3D printing technique. These characteristics include favorable physicochemical properties, biocompatibility, biodegradability, non-toxic degradation products, and antibacterial feature as well as their capacity for carrying cells and drugs for the recovery of soft and hard tissues [8,9,10,11,12]. Additionally, CS has great possibilities to enhance cell adhesion and viability, and also a substantial influence on the reproduction of damage tissue.

**Figure 2:**
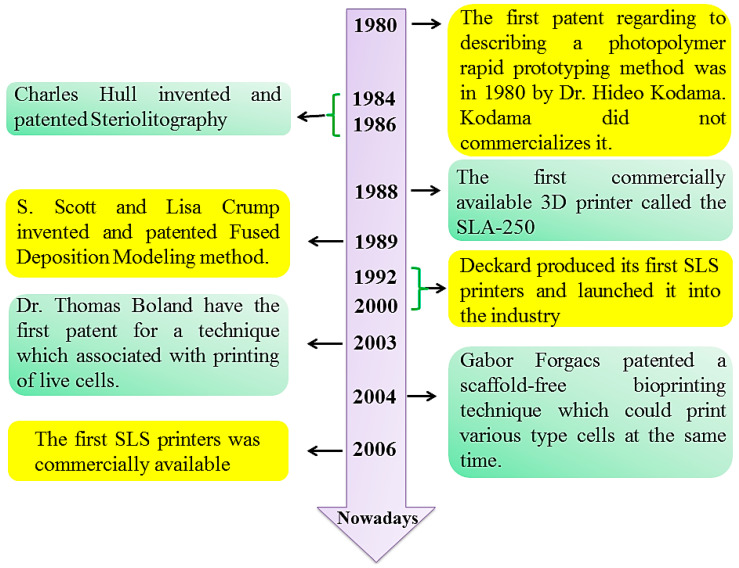
3D printing methods historical background.

On the other hand, many studies have made an effort to fabricate CS-based constructs by utilizing several conventional methods. However, limitations of these methods, such as low mechanical properties for electro-spinning, residual solvents in product and little control in pore size for solvent casting, energy usage, extensive timescale, the utilization of cytotoxic solvents, and the creation of tiny and abnormal-sized pores for freeze drying [13], have limited applications for thermoplastic components in phase separation, while closed pore structures for gas foaming have raised the necessity to look for another method of fabrication [11,13,14]. To overcome these limitations, a new technique called 3D printing or additive manufacturing has emerged. Appealing manipulations to produce personalized and patient certain designs, capability to duplicate, and the potential to prognosticate are the main advantages of this method. Furthermore, bio-printers offer a possibility to print the cell-laden matrices intended for productive reconstruction [11]. Additive manufacturing three is founded on the theory of layered production through which the components tend to be simply overlapped layer by layer. This system might be employed for rapidly prepared materials along with any complicated design by precisely gathering the components utilizing solid modeling based on a computer aided design (CAD) model [15,16]. Stereo-lithography (SLA), powder bed fusion (selective laser sintering (SLS), selective laser melting (SLM)), binder jetting, or indirect 3D printing, sheet lamination, directed energy deposition, material extrusion, and material jetting are the various types of additive manufacturing (AM) [17,18,19,20,21,22,23,24,25,26,27,28]. The 3D printing approaches developed in recent years can be traced back to the 1980s when the earliest SLA techniques were presented by Charles Hull [26,29], and in 1988, 3D systems launched from the commercial perspective with the accessible 3D printer, the SLA-250 [29,30,31,32,33,34,35] as shown in Figure 2.

The 3D printing continues to be employed in a broad range of health-related settings. One such setting is personalized pre-operation therapy which is intended for preoperative preparation and personalized operative equipment and prostheses. Adhering to a medicinal therapy, 3D printing is important in confirming the results attained by the affected person. Another one is examining various gadgets in particular pathways; an obvious illustration is the duplication of various vascular designs to examine the usefulness of a cardiovascular process utilized to deal with peripheral and cardiac artery illness. Enhancing healthcare education [26,36,37,38,39,40,41,42,43,44] is another 3D printing application, as certain 3D-printed patient types have shown that they might improve functionality and promote quick learning. The affected person education can be mentioned as another applicable advantage of these methods as patient-based care creates individual education among the major concerns for nearly all health-related suppliers. Furthermore, the 3D printing enables the creation of implantable tissue by bio-printing. Also, the 3D printing of drugs containing inks as a customized drug delivery can be done where the powdered drug layer to speed up its dissolution in comparison with the typical pills. Finally, the 3D printing could stand for a possibility to save life, decreasing the waiting list of affected individuals who require transplantation [26]. Despite all the advantages and the progress made in tissue engineering, it seems that creating whole organs remains a real challenge which has not been overcome yet.

**Figure 3:**
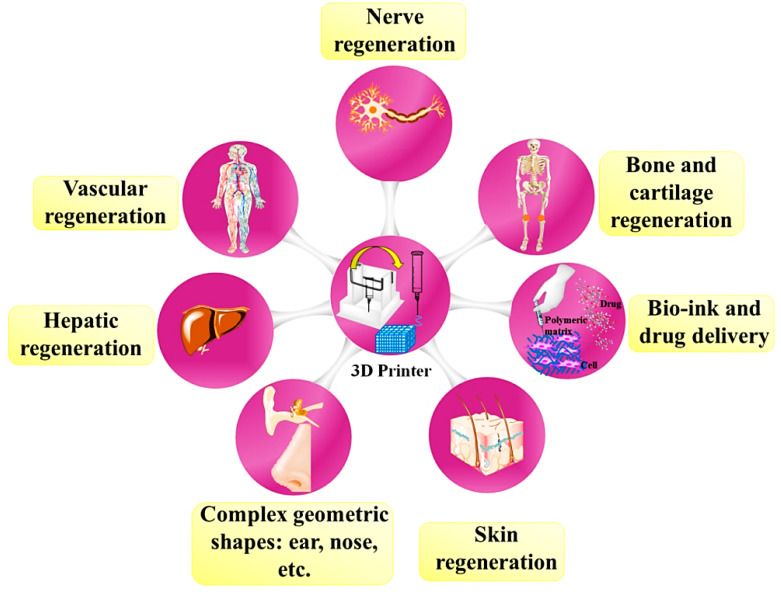
Various applications of 3D printed chitosan constructs.

Many studies have been performed to review the potential of CS and its derivatives for medical applications [36,37,38,39,40,41,42,43,44], but it is worth mentioning here that one of the most prominent differences between the present review and the others is that CS constructs created via 3D printing methods are reviewed specifically. In addition, for first time recent developments of methodology and the capabilities of 3D printing CS-based polymer are highlighted. Ultimately, essential restrictions are outlined to inspire the upcoming studies regarding 3D printing of CS-based polymers. Figure 3 shows various 3D printing applications for the preparation of different structures, including bone, cartilage, nerve, skin, vascular regenerations, and liver repairing as well as their capacity for carrying cells and drugs in order to recover soft and hard tissues. Nevertheless, nearly all 3D printed polymers products are employed as conceptual prototypes instead of practical components, considering that neat polymers constructed by 3D printing usually suffer from insufficient strength and performance as completely practical and load-bearing components. This kind of disadvantage limits the broad commercial applications of 3D printed polymers. The purposes of this figure are to determine a number of possible 3D printing purposes in various areas and to boost product development in a sustainable manner.

## 2. 3D Printing Methods

**Figure 4:**
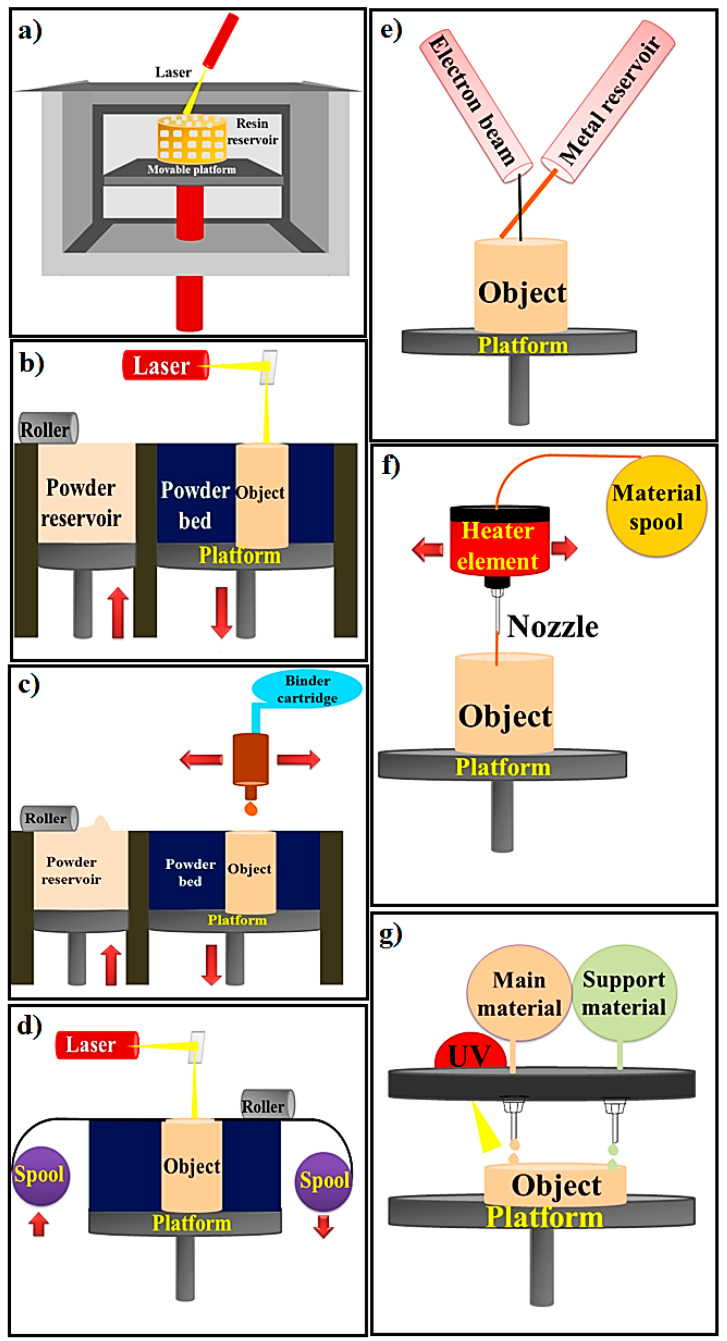
Various types of printing methods (**a**) Stereo-lithography, (**b**) Powder Bed Fusion, (**c**) Binder Jetting, (**d**) Sheet Lamination, (**e**) Directed Energy Deposition, (**f**) Material Extrusion and (**g**) Material jetting [18,19,21].

Numerous approaches are associated with the 3D printing, every one of which is displayed by one or more industrial technologies concept, as demonstrated by the ASTM International [45]. All the techniques displayed in Figure 4 [18,19,21] which are listed in Table 1 reveal the details regarding the technologies included, the components utilized, the clinical applications associated with every single method, and the positive aspects and drawbacks of each approach [26].

Regarding the, SLA method, it should be stated that it could be categorized based on build platform motion (There are two techniques that fall under this classification; namely, top-down and bottom-up) and laser movement (There are two types of techniques that are normally applied: projection-based stereo-lithography (PSL)) and scanning-based stereo-lithography (SSL) (see Table 1) [18]. The top-down method has a number of benefits in comparison with a bottom-up method. Initially, it is safer and more reliable to apply due to the fact that the laser is enclosed within just the printing unit. Therefore, the user is not subjected to the laser beam. Next, the curing procedure is performed in a closed atmosphere to protect against oxygen prohibition throughout photo-polymerization. Subsequently, recoating of the liquid resin employing a roller is not needed due to the fact that refilling occurs routinely with the help of gravitational pressure. Consequently, a top-down strategy may create softer printed components as a result of the complete contact between the liquid resin and the even base surface of the tank [18]. Stereo-lithography employs photopolymers which could be cured by UV laser. A UV-laser is normally manipulated in a preferred route to shoot in the resin tank, and the photo-curable resin could polymerize into a 2D patterned layer. Then each layer is cured, the system lowers, and an additional layer of uncured resin is prepared to be designed. Common polymer materials employed in SLA tend to be acrylic and epoxy resins. Comprehending the curing responses taking place throughout polymerization is essential to manage the level of quality of ultimate printed components [12,18,46]. Strength of the laser power, scan rate and timeframe of exposure has an impact on the curing period and printing quality. Photo-initiators and UV absorbers could be incorporated with the resin to handle the degree of polymerization. The primary benefit of SLA printing systems is the capability to print components with a high quality. Furthermore, considering that SLA is a nozzle-free approach, the problem of nozzle clogging might be prevented. Regardless of these positive aspects, the high price of this method is a primary issue for commercial plans. Feasible cytotoxicity of residual photo-initiator and uncured resin is an additional issue [12,46]. PSL prints every single layer together with a single shot of laser exposure through generating designed laser lights and SSL scans the surface of each one layer to produce designs. Therefore, PSL is appropriate for higher quality printing of small components as a consequence of the restricted size of the designed laser light. SSL, alternatively, is great for big size printing at the price of quality. PSL likewise offers a faster printing period in comparison with SSL since every single layer is printed in an individual shot [12,46]. Lately, the experts have designed a scanning-projection-dependent stereo-lithography (SPSL) which often couples PSL with SSL. SPSL employ a digital micro-mirror device (DMD), which is employed to generate designed laser lights in PSL and, by shifting the DMD, designed laser lights are also able to scan the resin surface area to permit big size printing. In the next approach, powder bed fusion (PBF)-centered systems, in which thermal energy selectively fuses regions of a powder bed. Selective laser sintering/melting (SLS and SLM), direct metal laser sintering (DMLS) laser, and electron beam melting (EBM) are the primary representative systems of PBF-centered methods. In the PBF approach, DMLS is an AM or rapid prototyping (RP) method which employs metal powder and also a great power laser to jointly sinter a useable component [12,22,46]. This kind of approach is suitable for generating extremely compacted components although in order to attain gas or pressure rigidity, post-treatment is typically needed. A large number of trade names including laser sintering explain the same method but not a different approach. The method is highly comparable to the current AM approach known as selective laser sintering (SLS). Both SLS and DMLS are basically the same approach; however, rather than employing polymers or coated metal powders in the case of SLS, DMLS utilizes uncoated pre-alloyed metal powders as the sintering material [12,22,46]. The EBM system utilizes a heated powder bed of metal in a vacuum which is subsequently melted and created layer by layer by utilizing electron beam energy (EBE) origin comparable to that of an electron beam welding (EBW)/electron microscope (EM) [22].

The third one, binder jetting, occasionally known as (indirect) 3D printing, is outlined by ASTM as an additive manufacturing technique in which a liquid bonding agent is selectively precipitated to bind the powder components. Table 1 demonstrates the key elements of a binder jetting (BJ) device, including the powder roller, powder stock, build platform, powder bed, binder cartridge, and inkjet print-head [24,47]. The printing approach functions as follows: initially, the powder roller develops a thin film of powder from the powder stock onto the develop system, creating the powder bed. Subsequently, the print-head jets binder (BJ) onto places which have already been outlined through the layer pattern of the 3D data file and afterward the powder particles in the selected parts are attached with the nearby particles. After one layer is completed, the created system is diminished by an outlined height and after that a fresh powder layer is propagated onto that completed layer. These actions are duplicated until the entire component is eventually completed. The completed component, known as the green component, is subsequently split up from the loose powder. The post-handling actions are curing, debinding, sintering, and recommended compaction [24,47].

Sheet lamination process as the fourth method consists of ultrasonic additive manufacturing (UAM) and laminated object manufacturing (LOM). LOM is one of the primary commercially accessible AM techniques, which can be dependent upon layer-by-layer cutting and lamination of the sheets or rolls of components [19,21,24,47]. Effective layers are accurately cut, implementing a technical cutter or laser, and tend to be then attached together (form-then-bond) or the other way around (bond-then-form). The form-then-bond technique is specifically valuable in the case of the thermal bonding of ceramics and metallic components that makes it possible for the building of inner characteristics by eliminating unwanted components prior to bonding. The unwanted components after cutting are remaining intended for assistance and right after completion of the procedure they are usually eliminated and reused. LOM could be employed for a wide range of components such as polymer composites, ceramics, and paper and metal-filled tapes. Post-handling like high-temperature treatment could be needed based on the kind of components and preferred characteristics. Ultrasonic additive manufacturing (UAM) is a novel subcategory of LOM which is a combination of the two methods including ultrasonic metal seam welding and CNC milling in the lamination process [19,21,24,47]. UAM is the only AM technique that is certainly capable of building metal constructions at low temperature. LOM is actually employed in numerous industries, including paper production, foundry sectors, electronics, and smart setups. Smart setups are categorized as constructions that may be multi-tasking with several sensors and processor chips. In contrast to traditional techniques, UAM may identify cavities in the framework according to the incorporated computer model for loaded electronic equipment, sensors, pipes, and additional characteristics. Electronic equipment is often printed in the identical lamination approach of UAM applying direct write systems. LOM might result in a lessening of tooling expense and production period, and is one of the greatest AM approaches for bigger constructions [19,21]. Nevertheless, LOM offers unfavorable surface quality without post-handling and its dimensional precision is less compared to the powder-bed methods. Furthermore, eliminating the unwanted components of laminates following the creation of the item is time-consuming compared to the powder-bed approaches. For this reason, it is not suggested for complicated patterns [19,21].

The fifth method, direct energy deposition (DED), is actually employed for the production of high-performance super alloys. DED works by using an origin of energy (laser or electron beam) which is often specifically concentrated on a small area of the matrix and is additionally employed to simultaneously melt the feedstock components (powder or wire). The melted components are subsequently precipitated and merged directly into the melted matrix and solidified right after the motion of the laser beam [19,21]. The DED and SLM approaches can be distinguished from each other by the fact that no powder bed is applied in DED and the feedstock is melted prior to precipitation in a layer-by-layer trend compared to FDM however by having an incredibly greater quantity of energy intended for melting materials. Hence, this might be valuable for filling cracks and retrofitting constructed components for the purpose of the application of the powder-bed approach. This approach permits for each multiple-axis precipitation and several components simultaneously. On top of that, DED could be combined effortlessly with traditional subtractive techniques to accomplish machining [19,21]. This approach is typically employed with titanium, inconel, stainless steel, aluminum, and the associated alloys in the aerospace industry. DED is often applied for big parts with low difficulty and complexity and also for fixing bigger parts. DED may lessen the production period and price, and offers outstanding mechanical characteristic, manipulated microstructure, and precise composition adjustment [19]. The next method is the fused deposition modeling (FDM) or fused filament fabrication (FFF) approach. FDM employs a continuous thermoplastic filament as the printing component to form great structures and scaffolds [26]. In this method, it functions by melting the plastic filament in the extruder nozzle head and accurately deposit ignite on a 3D platform to form a 3D structure based on the 3D data supplied to the printer. FDM procedure requirements support constructions pertaining to numerous bio-applications with affecting geometries. FDM procedure is an extremely precise and reliable procedure which is studio-friendly [26].

The last one, material jetting (MJ), is a drop-on-demand inkjet approach with a great spatial resolution that deposits one drop of ink at a period through jetting and subsequently a reaction or evaporation is triggered by an external energy origin (e.g., ultra-violet light, heating) to form a solid framework [48]. Whereas numerous aspects including liquid density or surface tension and print head or nozzle design might have an impact on the outcomes, the restriction on rapid viscosity will become the most challenging feature for droplet creation in material jetting. The transformation of the liquid material droplets to solid geometry must be carefully controlled. Material jetting is based on a phase transformation of the printed components. Types of phase transformation settings utilized in current printing systems tend to be: solidification of a melted material (e.g., wax, solder), evaporation of the liquid part of a solution (e.g., a number of ceramic methods), and healing of a photopolymer (e.g., Objet, ProJet machines) or several other chemical responses. Though the common issues of components jetting for 3D manufacturing are recognized, one can find many features which might not be effectively or completely comprehended [26,48]. Open investigation concerns are readily available in virtually all phases of the printing approach-droplet creation, precipitation control, and multilayer deposition. In the case of polymer jetting, one of the most suitable restrictions to tackle is that of droplet creation [48,49]. Due to the fact that devices designed for inviscid components are being employed for these purposes, several accommodations and restrictions are presently available. Users typically deal with this by adjusting the components to fit the specifications of the present equipment. Nevertheless, if the technique of droplet formulation might be improved as an alternative, this could possibly permit the precipitation of a broader variety of components [49].

## 3. Chitosan (CS)

When the level of deacetylation (DA) of chitin gets to around 50% according to the source of the polymer, it becomes soluble in aqueous acidic media and is known as CS. The level of DA offers the glucosamine to N-acetyl-glucosamine ratio and is commonly categorized in the spectrum of 50%–95% [50,51,52]. The molecular weight of commercially accessible CS ranges from ~100 to 800 kDa according to the origin and handling parameters. Each of these, i.e., the level of DA and the molecular weight (Mw) presented a significance effect on additional physicochemical characteristic of CS such as solubility, crystallinity, and degradation [50,51,52]. As an illustration, chitin (0% DA) and complete (100%) DA chitosan achieve the highest crystallinity in contrast to CS with moderate degrees of DA which is semi-crystalline. Also, the greater degree of DA resulted in greater degradation rates.

CS is insoluble in neutral and basic solutions (pH > 7). However, primary amines on deacetylated subunits of CS have a pKa of 6.5 and hence the CS types are water-soluble salts in each organic and inorganic acids [50,51,52]. The solubilization takes place through protonation of the -NH_2_ function on the C-2 placement of the D-glucosamine replicate unit, by which the polysaccharide is transformed into polyelectrolyte in acidic solution. The deacetylation, typically carried out in the solid state, provides an irregular structure because of the semi-crystalline feature of the original polymer. Assessment of the part of the protonation of CS in the presence of acetic acid and hydrochloric acid (HCl) on solubility revealed that the level of ionization relies on the pH and the pK of the acid [8]. The degradation behavior of CS in living organisms relies on the degree of DA and Mw. CS is usually absorbed by enzymes that hydrolyze linkages between glucosamine-glucosamine, glucosamine-*N*-acetyl-glucosamine, and *N*-acetyl-glucosamine-*N*-acetyl-glucosamine units. In the body system, CS is absorbed because of the activity of lysozyme and bacterial enzymes present in the colon. Similarly, the absorption of CS is often performed by chitosanase, chitin deacetylase, and β-N acetylhexosaminidase [51]. CS utilization in drugs is extremely varied. However, tissue engineering (TE) and wound dressing (WD) tend to be its two primary applications [53,54,55,56,57,58,59,60,61,62,63,64,65,66,67]. It was exhibited [68] that CS might be employed to hinder fibroplasia in wound healing and to enhance cell growth and attachment. Fibers manufactured from chitin and CS are generally valuable as biodegradable sutures and wound dressing components.

## 4. Chitosan-Based 3D Printed Construct for Hard Tissues Application

CS naturally has inadequate mechanical property. So, it is usually employed for hard tissue reproduction if its mechanical characteristic might be enhanced with inclusion of biomaterials including hydroxyapatite (HA), bioactive glass ceramic (BGC), etc. [69] or carbon-based additives including graphene [70], graphene oxide [71], and carbon nano-tube [72]. Occasionally, besides the reinforcement agent, inorganic compounds are utilized to generate a greater interaction between CS and the reinforcement agent, for example the 3D printed Maleic anhydride-grafted polylactide (PLA-g-MA) and CS composites in the Sano study [73]. In this research, regardless of the fact that all CS incorporated specimens with and without maleic anhydride portrayed appropriate cell response and antimicrobial performance, the PLA-g-MA/CS materials displayed greater mechanical characteristic in comparison to the PLA/CS composites. This influence was associated with a higher compatibility between the grafted polyester and CS.

### 4.1. Bone Regeneration

Bone tissue engineering (BTE) is an interesting area of tissue engineering (TE) which has attracted the attention of scientists and clinicians to create natural human bone (HB) by means of artificial devices. Bone, a key component of the HB, is a firm, tough, extremely specialized, arranged and compacted connective tissue [74,75,76]. It has several essential functions and is made from cells, organic and inorganic matrix. Similar to any other TE process, BTE involves a scaffold matrix with/without cells and biological cues for an effective result, i.e., bone reproduction. The biomaterials employed for building a bone scaffold matrix consist of polymers (natural or synthetic), natural-synthetic polymeric blends, and ceramics or polymer-ceramic composites [77,78,79,80]. One of the most broadly utilized polymers for bone reproduction is CS. Its cationic character is essential for BTE fields as CS might form polyelectrolyte complexes with anionic biological macromolecules [77,78,79,80]. Particularly, anionic GAGs including heparin and heparan sulfate modulate the action of numerous cytokines and growth factors crucial to bone reproduction. Therefore, CS treatment with GAGs or CS incorporated with GAGs in vivo might play a vital role in making use of growth factors to assist in bone creation [50,77,78,79,80]. A crucial characteristic of CS regarding TE is the simplicity with which it is usually functionalized. Reactive main amines and secondary hydroxyl groups existing on CS permit for the incorporation of the side groups, peptides or amino acids, most of which are usually critical for enhancing CS characteristic for BTE applications [50]. CS degradation in vivo takes place through the action of lysozyme leading to CS oligosaccharides which tend to be subsequently embedded into GAG or glycoprotein pathways, metabolic pathways or excreted. Despite the fact that CS matrices provide numerous positive aspects, they present poor mechanical characteristic and are unstable. Therefore, typically pure CS cannot be employed for bone repair. CS polymeric blends have evolved as greater options to improve the mechanical characteristic of the scaffold matrix and work as osteoconductive matrices [77]. Numerous researches can be found that have made use of blending of different natural polymers and ceramics including CS/Gel/HA printed scaffolds created with appropriate mechanical characteristics, osteoblast cell adhesion, viability, and growth [81]. Furthermore, in most of the reports on bone healing according to 3D printed CS structures, ceramics were incorporated into the composite [82,83,84]. In this regard, HA is one of the most common ceramics which is used for this application [82]. Likewise, in numerous researches CS is incorporated into the synthetic-based polymer, including PCL, PLLA [85,86,87,88,89,90,91], or synthetic polymers and ceramics are incorporated at the same time [92,93,94,95]. Even though synthetic polymers offer positive characteristics including cytocompatibility and sufficient mechanical performance, their hydrophobicity and the absence of particular cell recognition sites restrict their functional application. Thus, blending hybridized, synthetic, and natural polymers provides molecularly structured, bioactive characteristic and tunable mechanical property. By way of example, in a study by Dong et al. [85], in order to enhance the cell seeding productivity and osteoinductivity, an injectable thermo-sensitive CSG was embedded into a 3D-printed PCL scaffold by impregnating the PCL scaffold manufactured by FDM method, the first SFF in the pre-cooled CS/GP solution [85].

In addition, rabbit BMMSCs and BMP-2 were embedded into CS hydrogel (CSG). The key specifications of a perfect bone tissue scaffold should consist of macro-porosity and micro-porosity of around >100 μm and <20 μm respectively, interconnected open pores for cell migration, adequate mechanical performance, and suitable in vivo degradation duration to fit the healing rate of tissue in-growth [85]. According to the research, the average pore size of PCL scaffolds was around 325.2 μm and the pores of hybrid scaffold were successfully coated with CS hydrogel and reached to 62.4% which seemed to be in the appropriate range. The compression modulus of PCL scaffold was almost similar to that of the hybrid scaffold while the CM of the hybrid scaffold was considerably higher compared with pure CS gel [85]. The compressive strength of PCL and hybrid scaffold was around 6.7 MPa (comparable to cancellous bone tissue) and is significantly greater in comparison with other polymeric scaffolds fabricated from PCL. However, the results of in vitro degradation study by Dong et al. [85] pointed out a low degradation ratio for the PCL scaffolds, which happens to be a barrier for the formation of fresh bone. Then again, roughly 60% of CSG is degraded right after three weeks of soaking in PBS. Ultimately, the weight loss ratio of the hybrid scaffold amplifies steadily and is lower compared with CSG as a result of the presence of PCL. The fast degradation of CSG in the hybrid scaffolds permits fresh tissue creation and the constant release of incorporated bioactive molecules even though the remaining PCL is prepared to offer extra mechanical aid for the neo-tissue. A constant release of rhBMP-2 from CSG was noticed about 144 h throughout in vitro soaking. This verified the possibilities of CSG as osteogenic scaffold components. Likewise, several cells in gels and gel-embedded scaffolds were higher compared to the pure PCL scaffold. The ALP level of MSCs in CSG and hybrid groups was considerably greater compared with the PCL group at seven days, and the ALP levels preserved up to the point of 14 days. These results indicate that CS embedded into PCL scaffolds improved osteogenesis of BMMSCs for seven days, as opposed to pure PCL scaffolds. Keeping that in mind, at 14 days, CS and CS-embedded PCL constructs portrayed incredibly higher levels of (Alkaline phosphatase) ALP, (osteocalcin) OCN, and (collagen) COL1 compared with pure PCL scaffolds [85,92,93,94,95].

**Figure 5:**
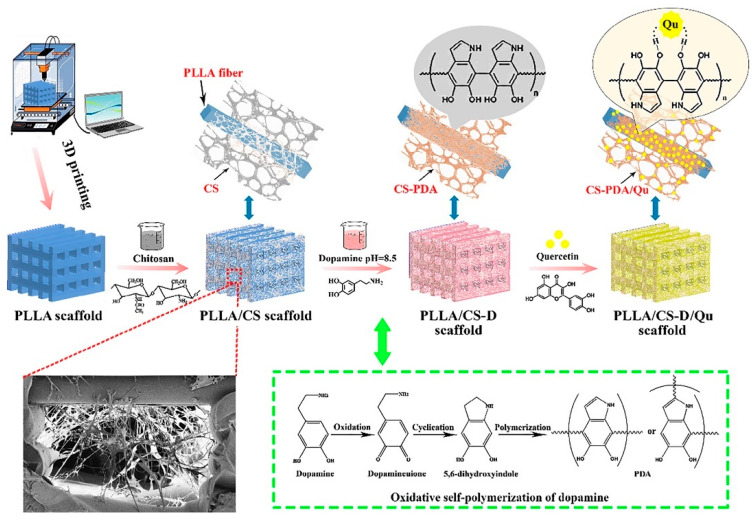
Schematic representation of the preparation process of the scaffolds [86].

The gene expression levels were comparable for both hybrid and CS gel specimens, indicating that CS is the primary component causing the improved osteogenesis and the PCL scaffold could not have an effect on in vitro osteogenesis. Nevertheless, the outcome could possibly vary when the hybrid scaffold is employed in vivo in which mechanical characteristic has a crucial role in the regulation of cell differentiation. By implantation of scaffolds in mice, ARS revealed just disperse calcium deposition in the CSG specimen and restricted calcium deposition on the PCL scaffold fibers. On the other hand, more substantial area of ARS was formed in the pores and fibers of hybrid scaffolds [85,92,93,94,95]. Furthermore, it was exhibited that the pore sizes >300 μm are generally helpful for improved fresh bone generation and the creation of capillaries. Small pores preferred hypoxic circumstances and would probably stimulate chondrogenesis in contrast to larger pores that result in greater angiogenesis and direct osteogenesis [85]. Hence, the FDM method was employed to selectively construct the PCL scaffolds of 300 μm pore sizes to fulfill the aforementioned qualification of bone scaffolds [85]. In a comparable examination, CS was blended with a synthetic polymer to repair the bone tissue (BT). The pure PLLA scaffolds had been printed through an FDM method and soaked into the CS solution and eventually the frozen scaffolds were removed and dried to attain the PLLA/CS scaffolds. Consequently, the PLLA/CS scaffolds loaded with Quercetin (C15H10O7, Qu) and dopamine (D) with the aim of enhancement of mechanical property and drug loading ability were obtained. The fabricated scaffold (PLLA/CS-D/Qu) presented great bone generation, antioxidant and anti-inflammatory characteristics, and compressive strength (15.06 MPa) (Figure 5) [86]. According to Dong et al. [85], the high compressive strength of the PCL/CS scaffold is attributed to the formation of a great hydrogen bond between the -C=O of PLLA and -NH_2_ of CS [86]. Taken together, the results illustrated the significant effect of CS in the enhancement of osteogenesis level, whereas poor mechanical properties limited the application of the CS polymer for 3D printing. However, after incorporation of HA [83,96], calcium phosphate (Ca-P) [84], laponite [93], and bioglass (BG) [97] into the natural polymers, the ability of CS scaffolds for 3D printing for bone repair was remarkably escalated. In addition to the mechanical properties, incorporating various types of materials into each other helps to achieve optimal rheology, the parameter which can influence the printability of the compounds used as the slurry for constructs made by 3D printing. As an illustration, the inks contained suspensions of Ca-P particles in CS acidic aqueous solution were employed by Caballero et al. [84] who implemented the robo-casting approach for preparing CS/Ca-P scaffolds. Their outcomes portrayed that the ink rheological properties might be tuned via alerting ink composition; specifically, more printable inks were attained with greater CS amounts (0.19 mol·L^−1^). In a similar study conducted by Chavanne et al. [83], 3D printed CS/HA scaffolds were manufactured with appealing mechanical properties, including high compressive strength in the range of 16.32 MPa. Some binders such as lactic acid (LA) with a concentration of 40 wt % were employed to provide more suitable viscosity, greater wettability, and shorter solidification period [83]. Despite the fact that bioactive HA and degradable polymers are generally appealing biomaterials, mechanical characteristic of 3D printing-based composite structures still remains a main problem. The expected mechanical characteristic could hardly be attained through high temperature sintering approaches, since CS would likely decompose over 220 °C. Among the primary complications revealed in their study is the insolubility of the prepared specimens which requires to be additionally enhanced to guarantee long-lasting mechanical stability in SBF [83]. Similar studies revealed the same results regarding the enhancement of 3D printed CS mechanical characteristic through the incorporation of HA into the combination [82,96]. Bioglass as a bioactive reinforcement agent was embedded into 3D printed CS bases by Dorj et al. [97]. Their results displayed that CS/10 wt% nBG scaffolds prepared via robo-casting presented a bimodal structure containing macro- and micrometers structures. In this method, the composite solution is quickly solidified when the specimens are soaked in a dry-ice cooled tank. Their fabricated scaffold (CS/10 wt % nBG) presented great bioactivity, good cells attachment and proliferation owing to the existence of nBG which caused the CS-based printed scaffolds helpful for BTE. Despite the suitable combination for apatite formation which is momentous for bone regeneration, and the appropriate results of cellular assay, this study lacked an evaluation of mechanical properties, which is a significant feature for bone tissue engineering [97]. Hence, it seems that further studies are needed to confirm the worth of CS/nBG for bone reconstruction.

### 4.2. Cartilage Regeneration

Cartilage degeneration takes place because of genetic abnormalities, trauma or disorder which could in turn need to have operative intervention, mainly replacement surgical procedures. Despite the fact that temporary relief might be accomplished, in long duration, different issues including pain and loss of extremity features persist [77,98]. Cartilage tissue engineering (CTE) is designed for making an entirely recovered, functional and scarless tissue that might transcend the presently accessible therapy modalities for cartilage deterioration. GAGs offer stimulate surroundings in cartilage reproduction [77,98,99,100,101,102,103,104,105,106]. CS has a structural resemblance with GAGs, and the hyaluronic acid (HA) existing in particular cartilages induces chondrogenesis and is used for CTE 3D polymeric containing IPFP-ASCs and TGFb3 and BMP6 which offered a base of stem cells to engineer 3D tissues for cartilage restoration in a study by Ye et al. [107]. In this context, it appears that, rather than traditional cubic scaffolds, it is considerably better to print the preferred combination in much more complex shapes including ears and noses. In this regard, ear-shaped hybrid scaffolds (CS and PEGDA) were manufactured through a stereolithographic approach utilizing a 405 nm laser by Morris et al. [108]. Mw of CS (50–190 kDa), feed-ratios, and photo-initiator were the crucial factors to determine the mechanical performance, cell response, and printability of the final 3D print specimen. Cell response of CS with mechanical robustness of PEG in scaffolds made these combinations appropriate for restoration of complex tissue geometries [108], including the ones from the human ear. In another study, Reed et al. [109] revealed extremely porous, hydrophilic CS-AL scaffolds manufactured in 3DP molds intended to produce articular cartilage and subchondral bone. In this relation, 3D printed constructs may possibly enhance aqueous solution uptake, blood uptake, and cell distribution. Due to the fact that clinical purposes of cell-based approaches are restricted as a result of the expense of keeping cellular constructs on the shelf, possible immune reaction to allogeneic cell lines, and scarce tissue, these acellular scaffolds which may stimulate endogenous influx and uniform distribution of native stem cells from bone marrow could be great candidate for cartilage reproduction [109]. In this connection, chitosan based constructs have been used for soft tissue regeneration frequently [110,111,112,113,114,115,116,117,118,119,120,121,122,123,124,125,126,127,128,129,130,131,132,133,134,135,136,137,138,139,140,141,142,143,144,145,146,147,148,149,150,151,152,153,154,155] which are further discussed in the following sections.

## 5. Chitosan-Based 3D Printed Construct for Soft Tissue Application

### 5.1. Nerve Regeneration

Chitin and CS are effectively examined for nerve reproduction applications because of physico-chemical and biological characteristics including biodegradability and biocompatibility. The adhesion of neuronal cells on the CS membranes accelerates the healing rate of the nervous system. In a similar examination, CS fibers exhibited a superior attachment and migration of Schwann cells (SCs) which reconstructed axons to the Bungner bands in the nervous system [77,110,111,112,113,114,115,116,117]. On the flip side, SCI is a disaster that may result in severe motor, sensory, and functional disorders. Implanting biomaterials are actually considered as optimistic approaches to recover neurological functionality [118]. As Bardakova et al. [119] exhibited, there are recognized works in which lyophilized CS scaffolds were effectively examined as applicants for spinal cord reproduction. As it was previously mentioned, such fabricated scaffolds including freeze drying offer one major disadvantage: an inadequate rigidity of the fabricated scaffold and the impossibility to significantly improve mechanical performance. Therefore, in their examination the two-photon-induced microstereolithography technique was employed to create 3D scaffolds from a photosensitive composition according to CS-g-oligo (L,L-lactide) copolymer. This copolymer was employed as the primary material of a photosensitive composition for creating hydrogel scaffolds [119]. The fabricated CS-g-oligo (L,L-lactide) was dissolved in 3 vol % acetic acid for a specific duration with additional treatment to attain 4.9 wt % of copolymer solution. Subsequently, PEGDA and biocompatible Irgacure 2959 were incorporated into the solution as the cross-linking agent and photoinitiator, respectively. Eventually, a 3D model was fabricated for the treatment of spinal cord accidental injuries [119].

Axon guidance is a vital aspect to consider for the fabrication of tissue scaffolds employed to increase nerve reproduction. In this regard, Zhu et al. [120] developed CS 2D grid designs implementing a DBRP approach for the manufacturing of scaffolds intended for escalating axon guidance in adult DRG neurons. Mixing of laminin–CS solutions was conducted under starrier condition at 2000 rpm for a short duration (10 min) in order to synthesize uniform 2D surfaces. In vitro examination demonstrated that DRG neurites on these patterns effectively expanded upon and followed the laminin-mixed CS pathways. In this context, incorporation of laminin could possibly enhance the affinity of neurons and neurites for a CS substrate and enhance, or even guide, neurite growth. Despite the fact that laminin combined with CS at low amounts (CSLN3 and CSLN0) might enhance neuron survival and adhesion, Zhu et al. [120] presented a fairly different result regarding of neuron cultures on smooth surfaces. In their study, minimal difference was observed in terms of neurite length when incubated on CSLN0 and CSLN3 specimens, whereas substantial difference was found regarding the CSLN6 specimens with higher amounts of laminin. This suggests that neuron cell body adhesion and neurite elongation could be dependent on the amount of laminin. The primary obstacle to the utilization of CS for axon guidance in nerve reproduction is that CS fails to have any particular bioactivity to interact with neurons. This issue is generally solved via material treatment approaches similar to what had been carried out in the study by Zhu et al. [120]. In another work, Col/CS scaffolds were prepared via two different methods including freeze-drying and 3D printing and subsequently implanted into the lesion made in rats. Compared with the Col/CS scaffolds (freeze-drying), 3D printed scaffold escalated the locomotor function. Diminished dormancy and amplified plenitude were both noticed in motor-evoked potential, which verifies the enhancement of neurological restoration. On the whole, 3D printed scaffold exhibited considerable therapeutic effects on the spinal cord of the rat model. Their in-vivo result provides a confirmation for the remarkable effect of fabrication strategy on product effectiveness for tissue regeneration [118].

**Figure 6:**
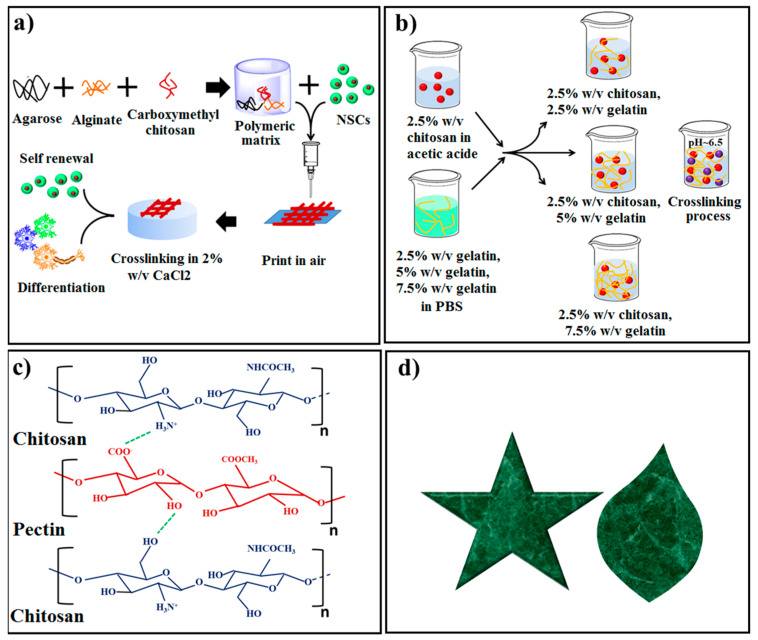
Soft tissue application of printed chitosan based constructs, (**a**) Schematic representation of hNSCc-laden AL-CMC-Ag bioink printing for nerve regeneration application, (**b**) Chitosan-gelatin preparation and cross-link process, (**c**) CS and PEC interactions to achieve wound dressing with a capacity of lidocaine delivery and (**d**) Starfish and leaf printed using chitosan ink referring to printability of chitosan ink at room temperature [121,123,138,155].

Direct-write printing of the stem cells (SCs) inside biomaterials provides a possibility for TE with reference to in vitro modeling. An initial illustration of creating neural tissue through printing hNSC which well differentiated promoting neuroglia can be found in a study by Gu et al. [121]. In their examination, various amounts of agarose (Ag) solution from 0.5 to 2.5% w/v were prepared in PBS and then AL was incorporated into the solution under stirrer condition for a short duration (30 min). Consequently, CMC was incorporated into the prepared solutions which were eventually cooled down to RT, prepared for mixing with hNSC and direct-write printing (Figure 6a) [121]. Porosity and permeability of gels were adjusted by CMC and the main role of CMC was to retain hNSC survival and this indicated the excellent cell-friendly behavior of CMC. In this context, the strength of AL/CMC gel was according to the range requirement for the human brain tissue (0.5–14 kPa).

### 5.2. Skin Regeneration

Skin traumas are typical clinical problems experienced by an incredible number of patients around the world and might lead to morbidity, impairment together with feasible death, with accompanying unfavorable side effects on the patients’ social and financial lives [122]. The recovery of skin integrity subsequent to an injury continues to be a crucial concern in medical practice such as settings including significant operations and incident and unexpected emergency wards [122]. Previously, the primary issue of wound treatment was to maintain the wound bed dry to prevent infection; nevertheless, the major purpose in the current wound administrations is the preservation of a balanced moist wound setting that permits the wound to heal in a regular fashion. The concept is that a moisture-rich environment assists to enhance the migration of keratinocytes by means of the augmentation of cell movements that will eventually facilitate the wound healing rate [122]. In recent decades, several natural and synthetic polymers have been used for wound healing such as CS [122,123], gelatin [124,125], hyaluronic acid [126], and poly vinyl alcohol [127]. Among these polymers, CSs are extensively being employed as skin regeneration components in dermal TE because of the property of hemostasis which improves tissue reproduction and induces collagen formation of fibroblasts and antibacterial activity [128,129,130,131,132,133,134,135,136]. CS in the shape of cotton fiber could improve wound healing by escalating the penetration rate of polymorphonuclear (PMN) cells at the wound site. In this regard, bFGF embedded CS demonstrated outstanding wound healing [77,137]. Likewise, CS-Gel blend is one of the combinations which are frequently used for skin regeneration [137,138]. In the examination conducted by Ng et al. [138] regarding 3D bioprinting, the negatively charged gel solution was added to the positively charged CS gel and subsequently various amounts of PGC in the range of 2.5%–7.5% were added to the final solution (Figure 6b) [123]. Polyelectrolyte Gel-CS was continued to be in a robust gel-state to enhance cellular adhesion and proliferation, along with other great characteristics including excellent printability [138]. Then, the polyelectrolyte Gel-CS bio-ink was incorporated into a sterile printing cartridge along with performing extrusion-based process. The outcomes revealed substantial shape fidelity and high fibroblast skin cells viability for the 3D printed specimens [137]. Intini et al. [139] utilized the freeze-gelation method coupled with modified CS solution containing raffinose fabricated FDM-3D printing approach. In their examination, two various 3D scaffolds with or without the CS-based layer were fabricated. After co-incubation of fibroblast and keratinocyte cells on the 3D scaffolds, it was found that all the 3D scaffolds containing CS presented greater performance including higher cell viability, cell attachment, and growth due to the presence of the CS film in the 3D printed scaffolds [139]. Furthermore, in vivo tests on the rat models of diabetes revealed that the employment of CS scaffolds to deal with wounds increases the reproduction of a tissue with an enhanced performance compared to the wounds healed with the commercial gauze, indicating the effectiveness of the CS scaffolds for the therapy of chronic dermal wounds. Typically, 3D printed CS scaffolds enhance the level of quality of the repaired tissue in comparison to the commercial gauze and spontaneous healing [139]. In the current scientific studies, genipin (GE) has typically substituted cross-linkers including glutaraldehyde to fabricate cross-linked CS for biomedical purposes because of the positive aspects including cytocompatibility, great chemistry and remaining less cytotoxic in comparison with glutaraldehyde, and pharmacological action, such as anti-inflammatory and antibacterial performance [122,140,141].

Hafezi et al. [122] fabricated 3D-printed films consisted of CS as film former and GE with GLY as cross-linker and PEG as plasticizer for wound dressing application. Their results revealed that the incorporation of GE into the CS matrix leads to the generation of hydrogel formation with a long term network structure, because of the creation of permanent chemical links which slowdown fast erosion through disintegration and ensure sluggish drug release as a consequence of slower rate of penetration via the structured matrix. Despite the similarity of film combination, the method of fabricating and the material used to model the drug release in Hafezi et al.’s [122] study (3D printing and fluorescein sodium) and Liu’s (cast in petri dish and dried in vacuum, Rhodamine B) were different. Nevertheless, the reason for less control over the drug release in Hafezi wt al.’s [122] study compared to the examination conducted by Liu [142] is the difference in the type of substance used to model the drug. In Hafezi et al.’s [122] examination, they applied an extremely soluble fluorescent dye which dissolves and penetrate quickly through the swollen dressing matrix and, consequently, more composition modification will probably be needed in the upcoming active components with moderate to low solubility, which is going to be supposed to manage more controlled drug release. Hence, there is no report for 3D printing method to have a negative effect on sustained drug delivery.

### 5.3. Vascular Regeneration

Cardiovascular diseases are essentially the most crucial reason of deaths. You can find a large amount of consumption of autologous bypass grafts for the remedying of the coronary artery problem. Nevertheless, the usages of these are restricted because of their particular hazardous features. Hence, this procedure (artificial grafts) is a required alternate choice for conquering vascular problems [143]. One of the suggested cure strategies is tissue engineering. Regardless of the development in TE, a number of concerns would certainly be required to be tackled for organ printing. The most essential concern is the incorporation of a vascular network, which is an issue experienced by the greater part of TE technologies [144]. CS is among the components which might be employed as the carrier of growth factor for the reproduction of tissue. CS stimulates fibroblasts to generate interleukin-8, which is included in the migration and spread of the cells [12,145,146,147]. In a study by Zhang et al. [144], a new bio-printing manufacturing method is established, in which vessel-like microfluidic channels are directly printed in complicated shapes without any requirement of pre/post procedures utilizing CS and AL. Biomaterial and its cross-linker agents had been individually incorporated straight into the coaxial nozzle system, where the solutions were linked, crosslinking was initiated, and subsequently the gel was created with a hollow channel. Afterward, the hydrogels containing 2%, 2.5%, 3%, and 4% CS were synthesized to print microfluidic channels [144]. Their results exhibited that the type and concentration of the hydrogel solution had significant effects on printability and mechanical integrity of the product. On the other hand, microfluidic channels supported cell viability and showed a potential for vascular networks developing. In a number of studies, in order to increase the bioactive properties, hydrogels have been incorporated into the polymeric matrix. Commonly, by addition of hydrogel, the construct elastic modulusand cell attachment and survival will be enhanced. In this regard, the study by Ulag and co-workers [143] can be referred to. In their study, PCL and CS with low Mw and hydrogels (H) were incorporated for preparing the constructs for the enhancement of cells adhesion and spreading owing to the hydrophilic characteristic of hydrogel. Their results showed that by escalating the hydrogel content, the elastic modulus of the constructs (PCL/7CS/5H) amplified and reached to 174 MPa. In this study, PCL/7CS containing the highest amount of hydrogel demonstrated the highest degradation rate which was due to the higher swelling ratio. Also, the best cell attachment and viability belonged to the PCL/7CS specimen in the presence of the highest hydrogel content (5 wt%). Hence, it seems that the PCL/7CS/5hydrogel is often implemented as a biomaterial for vascular TE [143].

### 5.4. Hepatic Regeneration

TE for complicated organ including liver encounters more issues; apart from great biocompatibility and biodegradability, the scaffold is required to maintain a variety of certain features including a stable 3-D spatial microenvironment to imitate the arranged structures of natural liver, and a pre-established vascular bed for adequate nutritional requirements and oxygen delivery, as well as extremely porous structures for organ reproduction [77]. Chitin and CS have found applications in liver tissue engineering due to biological properties. The reason for choosing chitin and CS scaffolds for hepatic reproduction is the existence of GAGs in chitin and CS structure, which are usually primary materials of the liver ECM. CS and its components with GAGs have regulated the functions of vascular endothelial and muscle cells [77].

In several studies, CS has been used in combination with poly caprolactone, collagen, gelatin, and heparin by conventional manufacturing methods such as electro-spinning and lyophilization for liver tissue engineering [148,149,150,151]. However, very few studies have been performed on CS constructs using the 3D printing method for liver tissue engineering application. Hence, it seems that this field needs further study. Jangkang et al. [152] fabricated CS–Gel hybrid scaffolds with well-oriented and extremely porous structures through rapid prototyping coupled with micro-replication and freeze-drying approaches. Their results demonstrated that the scaffolds have a high porosity (>90%) and a pore size of 100 μm which will improve hepatocyte growth.

## 6. Drug Delivery

Lately, CS is considered for pharmaceutical fields and drug delivery purposes in which consideration continues to be concentrated on its absorption-boosting, sustained release, and antibacterial performance toward specific bacteria [122,153]. In this context, for perfect wound dressing loaded with drug, the long release of drug (24–48 h) is typically useful for the patient as it eliminates the requirement to take out the dressing regularly. Even so, the number of the times which a dressing requires changing is affected by some other parameters such as the kind, size and depth of the wound [122]. Long and co-workers [123] examined the possibility of fabrication of 3D printed CS-PEC hydrogel loaded with various amounts of LDC drug from 2% to 10% w/w for wound dressing applications. The solution was injected into 3D printing syringes to generate the gel structure. Their results exhibited that the CS-PEC hydrogel loaded with 10% w/w LDC presented a great amount of porosity along with small pores as well as a high swelling ratio, implying its capability to taking in exudate and keeping moist for wound dressing (Figure 6c) [138]. Long et al. [123] also suggested that the occurrence of H_2_ bonds among CS and PEC leads to the higher orientation of the polymer chains, and freeze-drying approach effectively lessened the moisture amount in the CS-PEC hydrogel. The drug release pattern exhibited that CS-PEC hydrogel loaded with various amounts of LDC presented the release of LDC in a controlled fashion within 5 h, while rapid release in the initial time may offer powerful pain relief [123].

## 7. Bio-Inks

Bio inks are generally the kind of materials employed for numerous purposes in 3D printed-scaffolds. In this regard, printability, fidelity, viscoelasticity, expense, and cross-linking duration are some of the crucial factors relevant to the choice of bio-inks. The presence of the cells inside the ink to utilize a bio-ink provides the possibility to print 3D structures which are often implanted into impaired bone tissue to enhance cell responses [82,154,155,156,157,158,159,160,161]. CS can be used as a bio ink either in pure form or in combination with other materials, especially other polymers [154,155]. Wu et al. [155] prepared the 3D-ink printing of CSat room temperature where the CS inks were fabricated via dissolving in acidic mixture prior to the 3D printing (Table 2). In this context, the prepared inks were extruded under pressure and subsequently the 3D printed specimens with starfish shapes were manufactured at ambient temperature (Figure 6d) [155]. The specimens presented high mechanical strength (around 97 MPa) in dry condition and great strain (around 360%) in wet condition.

Col and CS are extensively utilized as biomaterials for 3D-printing. Nevertheless, the use of Col/CS blends as bio-inks is yet hard to find. In an examination conducted by Heidenreich [154], various hydrogels in the form of Col/CS were synthesized to print scaffolds (single-layered) for TE applications owing to its appropriate viscosity, printability as well as concentration (Col/CS ratio). The fabricated bio-inks were stable for 44 h in PBS and did not induce any toxicity to the NIH-3T3 cells. The results also indicated that the concentration of Col in the Col/CS solution has a significant effect on viscosity. In Heidenreich’s study, further incorporation of Col into the solution increases the viscosity, enhances cell viability, cell attachment and alters the morphology of the cells [154]. According to Table 2, in another study, a derivative of CS, carboxymethyl chitosan was used in combination with gelatin and sodium alginate as bio-ink containing bone mesenchymal stem cells for 3D bio-printing [156]. The Gel/sodium alginate/CMCS hydrogel exhibited great equilibrium water content and suitable mechanical properties and antimicrobial activity. Generally, CS and its derivatives are favorable choices for drug and cell delivery; nevertheless, these are used in combination with other synthetic or natural polymers and ceramics which makes them compatible with the target tissue features.

## 8. Benefits, Limitations, and Future Prospects

Three-dimensional bio printing is a new manufacturing approach for the accurate placing of biological components, cells, and biomolecules in the scaffolds. Altering manufacturing parameters makes it possible for stimulating natural tissues with tailored biological characteristics appropriate for the recovery of tissues. The advantages of CS are actually broadly described; among the major positive aspects of CS, one can mention the capability to modify properties such as the degradation rate by altering the degree of deacetylation and it’s Mw [157,158,159,160,161,162,163,164,165,166,167,168]. Additive components are regularly incorporated into CS in an effort to enhance its printability, fidelity, or to fabricate new cell-laden matrices.

**Figure 7:**
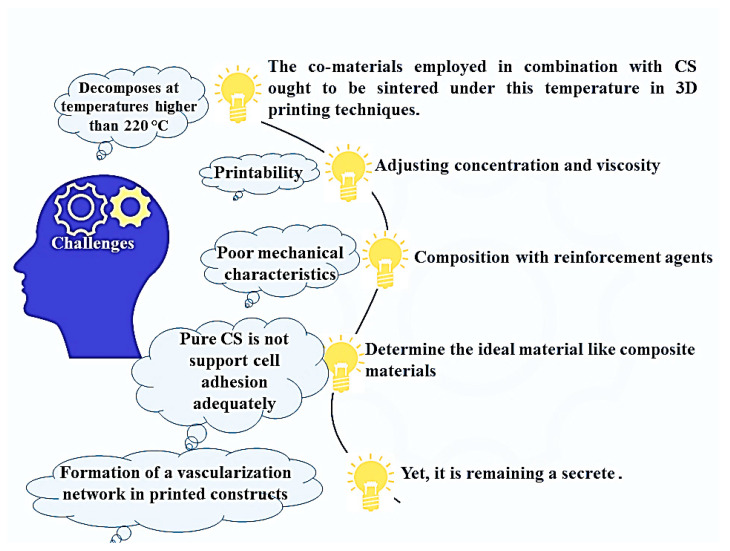
Challenges and solutions to overcome for chitosan 3D printed constructs.

However, it is worth noticing that CS decomposes at temperatures higher than 220 °C, thus the co-materials employed in combination with CS ought to be sintered under this temperature in 3D printing techniques. Moreover, pure CS is not going to really aid adequate cell adhesion; hence, it is vital to determine the ideal material (Figure 7) [167]. Nevertheless, its limitations can be in a wide range considering the type of different methods and their performance strategy. For instance, selecting the best binder for 3D printing is considered to be one of the difficulties for the Binder Jetting approach [11]. In general, selection of printing variables including dispensing pressure, dispensing speed, and preliminary height of dispensing tend to be the major issues in AM. These types of variables rely on the solution viscosity; hence, they are varied for every structure and concentration [96]. Occasionally, long cross-linking duration or gelation rate, shape instability, and poor mechanical characteristics are actually noted as the drawbacks of 3D printed specimens [169]. Among the most important issues of 3D printing is designing a purposeful organ full of vascular lumens in various dimensions. Almost all kinds of cells require a network of vessels to gain access to nutrients and oxygen and expel the unwanted products. For this reason, the formation of a complete complex vascularization network in the printed specimens has remained a challenge [170]. Ultimately, the capability to manufacture complex micro- or nanostructures through naturally derived hydrogels is important for biomedical purposes which are in turn met by the 3D printing approach. Nevertheless, accurately controlled architectures of soft hydrogels tend to be challenges to be overcome because of their restricted mechanical properties [171].

3D printing has been revealed as an innovative technology, appealing to convert the traditional products manufacturing. Nevertheless, restricted materials with appropriate printability, stability of construct in SBF and mechanical characteristic continue to be the serious challenges that have to be conquered prior to extensive designing for products manufacturing in biomedical fields. It is worth noting that the electro-spinning approach has gained considerable attention from biomedical experts all around the world for manufacturing various polymers, particularly CS-based polymer scaffolds loaded with drugs for biomedical and wound dressing purposes [172,173,174,175,176,177,178,179,180]. Application of CS-based biopolymers through 3D [181,182,183,184,185,186,187,188,189,190,191,192,193,194,195,196] printing for biomedical purposes especially as bio-ink in an effort to fabricate complex tissues will probably be an upcoming pattern of 3D printing materials progression. However, CS and CS/Col designed bio-printed skin tissue has not already obtained the FDA approval, taking into account the similarity of constructed materials with some other permitted materials. For this reason, it might be reasonably effortless to obtain regulatory approval in the long term [197]. Multi-material 3D printing for the rapid and highly accurate generation of physical models directly from volumetric data stacks, developed recently, is another promising method [198,199].

## 9. Conclusions

Three-dimensional printing appears to be an advanced approach, appealing to convert the traditional preparation of products. Nevertheless, restricted environmentally friendly printing components along with the high level of quality of printing capabilities remain serious challenges that should be conquered before it could be extensively modified for product preparation in various fields. In this context, 3D printing of a CS-based biopolymer was reviewed and it was exhibited that, regardless of the extensive studies, considerable issues continue to persist in the preparation of hydrogels with ordered structures and sufficient mechanical strength and biological characteristics for stimulating native tissues. Therefore, additional research is required to obtain suitable properties including great physical property, controllable pore size, great cell response, compatible mechanical properties with the target tissue mechanical characteristics, and capability to manufacture specimens with complicated geometric shapes.

## Figures and Tables

**Table 1:** 3D printing methods and their raw materials, advantages and disadvantages.

3D Printing Method	Materials	Device Components	Manufacturing Process	Advantage	Disadvantage	Ref.
Stereolithography (SLA); Bottom-up SLA; Top-down SLA	A resin with photo-active monomers	Laser, Vat of resin, UV light, Platform	The SLA technique is classified to top-down and bottom-up (based on build platform movement and laser motion). The laser is utilized for initiating photopolymerization and converting liquid resin to solid shape via photocuring process.	Various applications, Printing living tissues, Having the highest resolution among other printing methods, SLA has the ability of making structures with a resolution of 20 μm or less, which is the highest resolution among other printing methods (with resolution of 50-200 μm)	Lack of monolithic mechanical structure due to layer by layer fabrication process. Time consuming process caused by low photopoly-merization rates	[18,25]
PBF (SLS, SLM, 3DP)	Metals and alloys, Limited polymers, Ceramic	Laser, Powder roller, Powder bed, Powder stock, Platform	The working method is to spray powder materials on the previous layers and laser is utilized for fusing powders together.	Good resolution, High quality	Slow printing rate, Expensive process, High porosity	[20,22]
Binder Jetting or indirect 3D printing	Metals, Polymers, Ceramics	Powder roller, Powder stock, Build Platform, Powder bed, Binder cartridge, and inkjet print head.	The binder jetting techniques is used for powders and powder layers binds together with adhesive. The powder is sprayed on the platform via roller. The head of print sprinkled the adhesive on top of the powder according to the structure designed by the computer. The platform comes down by the thickness of object’s layer. Next layer is made by spraying powder on the previous layer. The object is fabricated via powder and the liquid bounding.	Ceramics has more challenges to use by additive manufacturing than polymers and metals technologies due to high melting temperature; Hence, binder jetting can be a promising method to fabricate ceramic based materials. Cost effective, No shrinkage	Low mechanical properties	[24,47]
Sheet Lamination	Metals (aluminum, copper, stainless steel and titanium), Ceramic, and Composite	Laser, Platform, Mirror, Material spool, Cross hatched material, Support material,	UAM and LOM are two strategies of sheet lamination. The material is placed on platform and bonded to the previous layer by the adhesive materials. The designed structure is cut from the layer via laser. Then next layer is made.	Low shrinkage and residual stresses, Quick process	Difficulty of precision in the Z-dimension control, Lack of mechanical homogeneity in products because of utilizing adhesive in fabrication process	[21]
Directed Energy Deposition	A resin with photo-active monomers, Hybrid polymer-ceramics, Metals and alloys in the form of powder or wire, Ceramics and polymers	Electron beam, Metal Wire supply, Metal wire, Platform	The powder or wire is placed in the pool of melt which is glued to a lower part or layers via source of energy (laser or electron beam).	Cost effective and quick process, Favorable mechanical properties, Control on microstructure	Low accuracy and surface quality, Restrictions on printing complex geometric shapes with precise details	[19]
Material extrusion FDM and FFF	Plastics, Polymers	Material spool, Heater element, Nozzle, Heater element	Thermoplastic materials are melted and extruded and create layers by moving the nozzle according to the computer design.	Ease of use, Suitable mechanical properties	Filament required, Restriction of raw materials, Inability to print live cells	[26]
Material jetting	Plastics, Polymers	UV light, Elevator, Platform,	The MJM mechanism of action is similar to ink jet printer. Material jetting on platform is done (drop or continuous)	High accuracy, Low waste of material	Restriction of raw materials: polymers and waxes, Required support material	[48,49]

**Table 2:** The utilized material, method and physical, mechanical and cellular characterizations in 3D printed chitosan based construct.

Biobased-Material	3D Printing Method	Solvent	Printed Structure	Porosity, Pore Size	Mechanical Properties	Cellular Assay	Cell Type	Target Tissue	Ref
CS, PCL-DA and PEG-DA	RDMAM system	Benzene, acetone and acetic acid.	Multi-layer scaffolds	Pore size = 300 μm	PCL-DA/PEG-DA/CS 5% tensile strength = 0.75 ± 0.05, PCL-DA/PEG-DA/CS 10% tensile strength = 0.53 ± 0.04, PCL-DA/PEG-DA/CS 15% tensile strength= 0.29 ± 0.09 Elastic Modulus= 14.97 ± 3.99 kPa	Well cell viability and proliferation	L929 cells	TE	[16]
CS (6% w/v) and CS modified with raffinose	FDM	2% acetic acid	3D scaffolds	Feret diameter: scaffold without raffinose 10 ± 20 μm; scaffold with raffinose 3.5 ± 3 μm	-	Well cell adhesion and proliferation	Fibroblasts	Soft tissue engineering	[51]
PLA, CS and Maleic anhydride-grafted PLA (PLA-g-MA)	An extruder (by heating and melting)	-	(3D) printing strips	-	Tensile strength of PLA-g-MA/CS (20 wt%) ≈ 52	Well cell viability	Human foreskin fibroblasts	Biomedical material	[73]
CS, Gel and HA	FDM	2% acetic acid	3D scaffolds	Pore size ≈ 200–500 µm	-	Well cell viability and proliferation	MC3T3-E1 cells	BTE	[81]
AL, AL-HA, CS, CS-HA	The Fab@Home™ (The Seraph Robotics) open source RP platform Model	PBS, 0.1 M acetic acid	Scaffolds with disc shape (6 mm diameter × 1 mm thickness)	Average pore size of pure CS ≈ 200 μm and CS-HA ≈ 100 μm	-	Well cell viability, proliferation and osteogenic differentiation	MC3T3-E1 pre-osteoblast	BTE	[82]
CS, HA	Z-Corp, Z-510 Solvent/dispensing	Lactic acid, citric acid, acetic acid	3D scaffolds	Porosity = 37.1%	Compressive strength = 16.32 ± 2.8 MPa Elastic Modulus = 4.4 ± 2.1 GPa	-	-	BTE	[83]
CS, calcium phosphate	Robocasting	Acetic acid	3D scaffolds	Porosity = 22%	-	-	-	Filler for large bone defects	[84]
PCL, CS	FDM	0.1 M acetic acid	3D scaffolds	PCL/CS porosity = 62.4 ± 0.23% The pore size of PCL scaffolds = 325.2 ± 26.3 μm	Compressive strength ≈ 6.7 MPa	Well Cell viability, Proliferation and expressions of Osteogenic gene	Rabbit BMMSCs	BTE	[85]
PLLA, CS and bioactive Qu, PDA	3D printer (MakerBot Replicator Z18) via a FDM)	0.1% (v/v) acetic acid aqueous solution	Cylindrical scaffolds	-	Compressive strength of PLLA/CS-D/Qu ≈ 15 MPa and elastic modulus ≈ 0.140 GPa (dry condition)	Well cell attachment, osteogenic activity and good anti-inflammatory feature	MC3T3-E1 cell	BTE	[86]
CS, PVA and various ratio of HA (2.5, 5, 10, and 15 wt %) And BMP-2	Pushing of Hydrogel from the syringe (by computer controlling) and spraying the crosslinking agent	Acetic acid, distilled water	3D scaffolds	Pore size = 800 to 1300 μm	Elastic modulus of CS/PVA containing 15 wt% HA ≈ 91.14 MPa	Well cell viability and adhesion	hMSCs	BTE	[92]
MAG-Lp, MAC-Lp	Robocast-assisted deposition system	Acetic acid	3D scaffolds	Average pore size = 389 ± 58 µm based on horizontal, 385 ± 38 µm based on vertical for MAC-Lp.	Compressive strength ≈ 14–15 MPa for MAC-Lp	Enhanced osteoblast growth and biomineral formation	MC3T3-E1	Osteoblast growth	[93]
PLA, CS and HA	FDM	0.36% of acetic acid	3D scaffolds	Very large pore diameter ≈ 960 ± 50 mm, Porosity ≈ 60%	PLA/CS-HA modulus = 16.4 ± 2.5 MPa	Well cell viability and osteogenic differentiation	hMSCs	BTE	[94]
CS, HA	Robotic dispensing System Solvent/dispensing	Acetic acid/NaOH ethanol	3D scaffolds	Macropore = 400–1000 µm for CS scaffolds, macropore size = 200–400 µm for the CS–HA scaffolds	-	Well cell adhesion and distribution	Osteoblasts	BTE	[96]
CS, nBA	Robocasting	Acetic acid	3D scaffolds	Macro structure (hundreds of micrometers) and highly micro-pore = a few to 10 μm	-	Well cell adhesion and spread	MC3T3-E1 preosteoblastic cells	BTE	[97]
CS scaffolds + IPFP-ASCs + TGFb3 and BMP6	Extrusion printed onto a glass slide, immersion in bath of isopropyl alcohol.	Acetic acid	Scaffolds	-	-	A shiny cartilage-like tissue ‘cap’, positive staining of collagen I, II and cartilage proteoglycans	IPFP-ASCs	Osteochondral graft	[107]
Resin, CS and PEGDA	Stereolithography	1% acetic acid	3D printed ear scaffold	Pore size ≈ 50 µm	Elastic modulus ≈ 400 kPa	Long term cell viability and spreading	hMSCs	Complex tissue geometries, such as human ear	[108]
CS, AL	Uprint, Z402	Acetic acid	3D scaffolds	Pore size ≈ 100 μm pores	-	Improvement of cell suspension uptake	Mouse bone marrow stromal cells	CTE	[109]
Col, CS	A 3D bioprinter	1% acetic acid	3D scaffolds	Porosity = 83.5% pore size 60–200 μm	Compressive	Implementing	NSCs were obtained from embryonic brains at day 14	SCI	[118]
					strength of 3D-Col/CS = 345.20, 29.60 KPa and Compressive modulus = 3.82 ± 0.25 MPa	3D-C/C scaffold enhanced the number of biotin dextran amine fibers and led to smaller cavity and a more linear-ordered structure			
CS-g-oligo (L,L-lactide) copolymer and PEGDA as a cross linker	Two-photon-induced micro stereolithography	3 vol.% acetic acid	A truncated cylinder scaffolds	-	-	A high survival rate of cortical neurons and the formation of neural networks	Dissociated rat cortical neurons	NTE	[119]
CS, laminin	DBRP	Acetic acid	3D nerve conduit scaffolds	-	-	Laminin improves the viability of neurons grown and the length of neurite growth	Adult DRG neurons	NTE	[120]
Al, CMC and agarose	Direct write printing (Extrusion-based-3DBioplotter System)	PBS	3D scaffolds	-	-	Well hNSC expansion and differentiation	hNSC	NTE	[121]
CS, GE as a cross linker, GLY and PEG as plasticizer	A 3D printer with jet dispenser	0.5% v/v acetic acid	Film	-	-	Well cell viability	Human skin fibroblast cell	Chronic wound healing	[122]
CS, PEC	Extrusion-based 3D printing	0.1M HCl	A mesh scaffold model	-	Self-adhesion to skin with bioadhesion strength in the range of 86.5–126.9 g	-	-	Wound healing, local LDC release	[123]
Polyelectrolyte Gel, CS	A 3D bioprinter, (extrusion-based print-head)	Acetic acid, PBS solution	A 3-layered grid-like patterns	-	-	Well cell viability and proliferation, spindle-like morphology	Fibroblast skin cells (HFF-1)	STE	[137]
Polyelectrolyte CS, Gel	A 3D bioprinter, Biofactory	CS in acetic acid, gelatin in PBS	Multi-layered hydrogel construct	-	-	Well cell viability and proliferation, spindle-like morphology,	Naonatal human foreskin fibroblasts (HFF-1)	STE	[138]
CS	FDM	Acetic acid 2% (v/v) containing D-(+) raffinose pentahydrate	3D scaffolds with grid of orthogonal filament	Pore size ranges = from 4 to 9 μm	-	An early skin-like layer consisting of fibroblast and keratinocyte	Human fibroblast (Nhdf) and keratinocyte (HaCaT)	STE	[139]
PCL, CS	Materials extrusion, (by melting materials)	-	Vessel-like scaffolds	-	Elastic modulus for PCL/7 wt%CS/5 wt%H = 174 MPa	Well cell viability and growth	HUVEC cell	Cardiovascular diseases	[143]
Al, CS	A single arm robotic printing	Deionized water, 1.0 M acetic acid	Channels in form of hollow tubes	-	Maximum tensile stress = 5.65 ± 1.78 kPa and Young’s modulus = 5.91 ± 1.12 kPa	Well cell viability	CPCs	Vascular networks	[144]
CS and Gel hybrid, glutaraldehyde as a cross linker	Combining rapid prototyping, microreplication and freeze–drying	1 wt% acetic acid	3D scaffolds	Porosity = 90–95%, pore size = 100 µm	Compressive strength ≈ 264 ± 10.1 KPa	Well hepatocyte attachment and viability ≈ a bove 90% Well albumin secretion and urea synthesis	Hepatocytes	HTE	[152]
Col, CS	A bioprinter with two syringes	0.10 M acetic acid	Meshes design	Square holes of 4 mm on each side	-	No cell morphology change, Non-cellular toxicity	NIH/3T3 fibroblasts monolayers	TE	[154]
CS	Extrusion-based 3D printing	Acidic mixture (40 vol% acetic acid, 20 vol% lactic acid, 40 vol% distilledwater	30-layer scaffolds, starfish, leaf, and spider shapes	Pore size ≈ 220 µm	Maximum tensile strength ≈ 97 MPa (dry condition) and high strain at break ~360% in the wet condition	-	-	Inks for 3D Printing, tissue engineering, drug delivery	[155]
BMSCs-laden Gel, sodium alginate and CMC	Micro extrusion-based 3D printer equipped with z-axis-controlled ink reservoirs	water	3D scaffolds	-	Young modulus ≈ 120 MPa	Well cell viability	BMSCs	TE	[156]
CS	Direct printing of chitosan ink in air (Extrusion-based method) and partial hardening via solvent evaporation	Acidic mixture: 40 vol% acetic acid, 10 vol% lactic acid, and 3 wt% citric acid).	3D scaffolds	Microfiber networks, pore size ≈ 220 μm	Tensile strength ≈ 7.5 MPa	Well cell Survival and proliferation	L929 fibroblasts	Biomedical materialS	[161]

AL: Alginate; BTE: Bone tissue engineering; BMSCs: Bone mesenchymal stem cells; BMP-2: Bone morphogenetic protein-2; CS: Chitosan; CMC: Carboxy methyl chitosan; CPCs: Cartilage progenitor cells; CTE: Cartilage tissue engineering; Col: Collagen; DBRP: Dispensing-based rapid prototyping; DRG: Dorsal root ganglion; FDM: Fuse deposition manufacturing; GE: genipin; Gel: Gelatin, GLY: Glycerol; HA: Hydroxyapatite; hMSCs: Human mesenchymal stem cells; hNSC: Human neural stem cells; IPFP-ASCs: Infrapatellar fat pad adipose stem cells; MAC-Lp: methacrylated chitosan-laponite; MAG-Lp: methacrylated gelatin-laponite; nBA: Nano bioactive glass; NVE: Nerve tissue engineering; NSCs: Neural stem cells; PBS: Phosphate buffer saline; PCL-DA: poly (ϵ-caprolactone) diacrylate; PDA: polydopamine; PEC: Pectin; PEG: Poly ethylene glycol; PEGDA: Polyethylene glycol diacrylate; PLA: Poly (lactide acid); PLLA: poly (L-lactide); PVA: poly (vinyl alcohol); Qu: quercetin; RDMAM: Reflective dynamic mask additive manufacturing; RP: Rapid prototyping; SCI: Spinal cord injury.

## Abbreviations

LOM	Laminated object manufacturing
MAC-Lp	Methacrylated chitosan-laponite
MAG-Lp	Methacrylated gelatin-laponite
MJM	Material jetting multijet
nBA	Nano bioactive glass
NVE	Nerve tissue engineering
NSCs	Neural stem cells
OCN	Osteocalcin
PBS	Phosphate buffer saline
PBF	Powder Bed Fusion
PCL-DA	Poly (ϵ-caprolactone) diacrylate
PDA	Polydopamine
PEC	Pectin
PEG	Poly ethylene glycol
PEGDA	Polyethylene glycol diacrylate
PLA	Poly (lactide acid)
PLLA	Poly (L-lactide)
PSL	Projection based stereolithography
PVA	Poly (vinyl alcohol)
Qu	Quercetin
RDMAM	Reflective dynamic mask additive manufacturing
RP	Rapid prototyping
SAL	Stereolithography
SCI	Spinal cord injury
SCs	Schwann cells
SLA	Stereolithography
SLM	Selective laser melting
SLS	Selective laser sintering
SFF	Solid free-form fabrication
SPSL	Scanning-projection based stereolithography
SSL	Scanning-based stereolithography
3D	Three dimensional
2D	Two dimensional
UAM	Ultrasonic additive manufacturing
UV	Ultraviolet
WD	Wound dressing
AL	Alginate
ALP	Alkaline phosphatase
AM	Additive manufacturing
ARS	Alizarin Red Staining
BGC	Bioactive glass ceramic
BJ	Binder jetting
BMP-2	Bone morphogenetic protein-2
BMSCs	Bone mesenchymal stem cells
BTE	Bone tissue engineering
CAD	Computer aided design
CMC	Carboxy methyl chitosan
CNT	Carbon nano tube
Col	Collagen
CPCs	Cartilage progenitor cells
CS	Chitosan
CSG	CS hydrogel
CT	Computed tomography
CTE	Cartilage tissue engineering
DA	Deacetylation
DBRP	Dispensing-based rapid prototyping
DED	Direct energy deposition
DMD	Digital micromirror device
DMLS	Direct metal laser Sintering
DRG	Dorsal root ganglion
EBM	Electron beam melting
EBW	Electron beam welding
FDM	Fuse deposition manufacturing
FFF	Fused filament fabrication
GAG	Glycosaminoglycans
GE	Genipin
Gel	Gelatin
GLY	Glycerol
HA	Hydroxyapatite
hMSCs	Human mesenchymal stem cells
hNSC	Human neural stem cells
IPFP-ASCs	Infrapatellar fat pad adipose stem cells
